# Supplementary material for: Motor domain phosphorylation increases nucleotide exchange and turns MYO6 into a faster and stronger motor
Source: Nat Commun. 2024 Aug 7;15:6716. doi: 10.1038/s41467-024-49898-3 (PMC11306250; doi:10.1038/s41467-024-49898-3)
Supplement: Supplementary file 1 — Supplementary Information [file 41467_2024_49898_MOESM1_ESM.pdf]

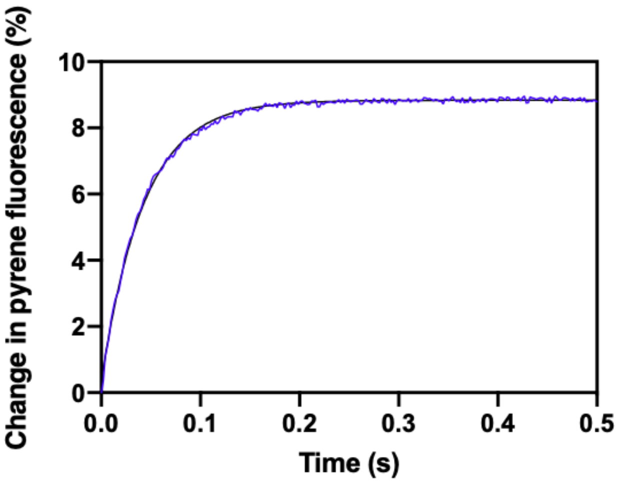


**Supplementary Figure 1. Biochemical characterisation of MYO6 phosphomutants.** Example stopped-flow trace to measure ATP-induced dissociatoin of pyrine-actin from MYO6 S267A. 2.5 mM ATP was rapidly mixed with 50 nM pyrene-actin.MYO6, and the increase in pyrene fluorescence was measured (blue). Least squares best fit to a single exponential is shown in black with $k_{obs}$ = 52.6 $s^{-1}$ and 8.3 % increase in pyrene fluorescence.

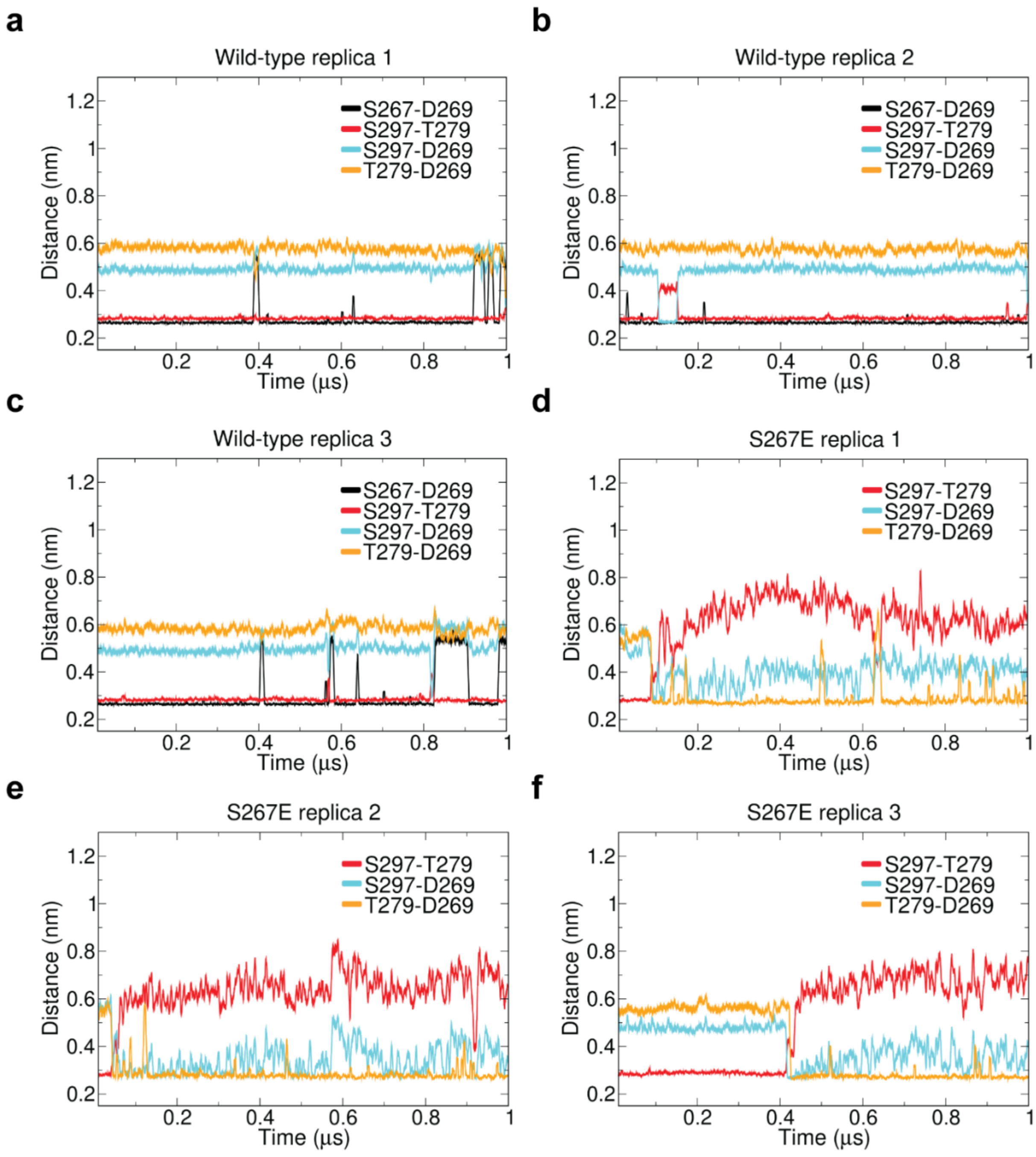


**Supplementary Figure 2. Time-dependent analysis of key contacts around residue 267.** Distance between the sidechain oxygens of the indicated residues in the WT (a-c) and S267E (d-f) simulation replicas.

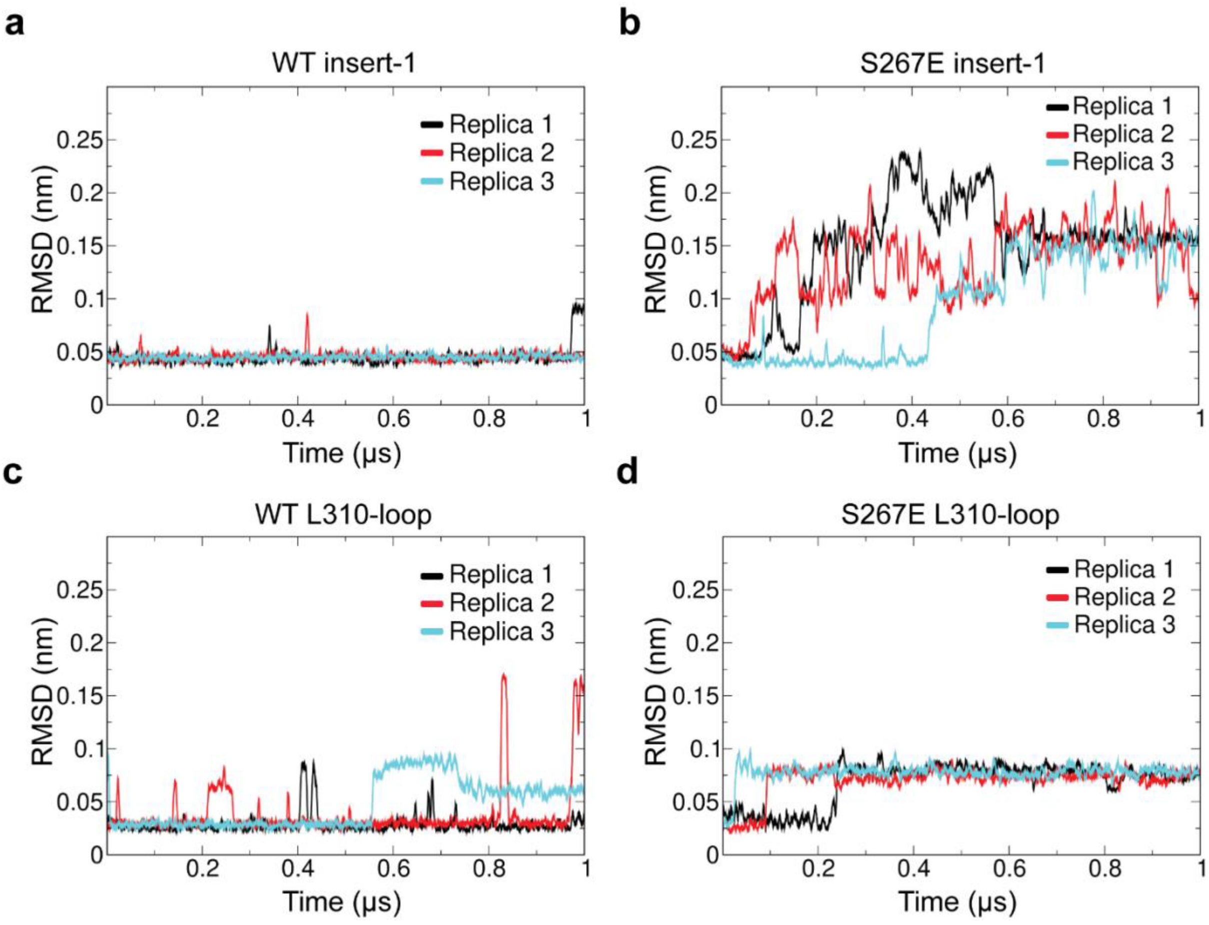


**Supplementary Figure 3. Time-dependent analysis of the root mean square deviation (RMSD) of insert-1 (residues 278-303) and L310-loop (residues 308-312).** RMSD in the WT (a), (c) and S267E mutant (b), (d) simulations. Replicas 1, 2 and 3 are shown in black, red and cyan lines.

| | WT | S267A | S267E |
|---|---|---|---|
| **ATP-induced dissociation from actin.MYO6 ($mM^{-1}s^{-1}$)** | | | |
| - $Ca^{2+}$ | 19.2 ± 1.6 | 23.9 ± 0.9 | 49.6 ± 2.6 |
| + $Ca^{2+}$ | 20.6 ± 0.8 | 22.8 ± 1.1 | 45.8 ± 3.8 |
| **ADP release rate from actin.MYO6 ($s^{-1}$)** | | | |
| - $Ca^{2+}$ | 3.5 ± 0.5 | 2.8 ± 0.3 | 16.1 ± 0.8 |
| + $Ca^{2+}$ | 2.9 ± 0.4 | 1.8 ± 0.3 | 5.9 ± 0.7 |

**Supplementary Table 1. Summary of transient kinetic data.** Errors reported are SEM from 3 replicates. Solutions were buffered with 25 mM KCl, 20 mM MOPS, 5 mM $MgCl_2$, 2 mM EGTA (-$Ca^{2+}$) or 2 mM Ca-EGTA (+$Ca^{2+}$). Experiments were performed at 20 °C.

| MYO6 MD system | Motor domain residues | Calmodulin residues | Number of water mole-cules | Number of Chloride ions | Number of Sodium ions | Number of Calcium ions | Salt con-centration (mol/L) | Box dimensions (nm) | Total number of atoms |
|---|---|---|---|---|---|---|---|---|---|
| WT | 3-815 | 3-147 | 100,971 | 287 | 299 | 4 | 0.15 | 14.6 x 14.6 x 14.6 | 318,759 |
| S267E | 3-815 | 3-147 | 100,986 | 287 | 300 | 4 | 0.15 | 14.6 x 14.6 x 14.6 | 318,809 |

Supplementary Table 2. Components of the MD simulation systems.
